# Relating aging and autophagy: a new perspective towards the welfare of human health

**DOI:** 10.17179/excli2023-6300

**Published:** 2023-07-31

**Authors:** Sougata Ghosh Chowdhury, Rachayeeta Ray, Parimal Karmakar

**Affiliations:** 1 Department of Life Science and Biotechnology, Jadavpur University, Kolkata-700032, India

**Keywords:** autophagy, aging, life span, misfolded protein

## Abstract

The most common factor that contributes to aging is the loss of proteostasis, resulting in an excess amount of non-functional/damaged proteins. These proteins lead to various age-associated phenotypes such as cellular senescence and dysfunction in the nutrient-sensing pathways. Despite the various factors that can contribute to aging, it is still a process that can be changed. According to recent advances in the field of biology, the ability to alter the pathways that are involved in aging can improve the lifespan of a person. Autophagy is a process that helps in preserving survival during stressful situations, such as starvation. It is a common component of various anti-aging interventions, including those that target the insulin/IGF-1 and rapamycin signaling pathways. It has been shown that altered autophagy is a common feature of old age and its impaired regulation could have significant effects on the aging process. This review aims to look into the role of autophagy in aging and how it can be used to improve one's health.

## List of abbreviations

AMPK AMP-activated protein kinase

ATM Ataxia telangiectasia mutated

CAMKK2 Calcium/calmodulin-dependent protein kinase kinase 2

CMA Chaperone mediated autophagy

CR Calorie restriction

DDR DNA damage response

DNA-PKcs DNA-dependent protein kinase catalytic subunit

EZH2 enhancer of Zest Homologue 2

IGF-1 Insulin-like growth factor 1

IRE-1 Inositol-requiring enzyme type 1

JNK1 c-Jun N-terminal kinase 1

LAMP2A Lysosome-associated membrane protein 2

NKX2-1 Nk2 homeobox 1 transcription factor

Nrf2 Nuclear factor erythroid 2-related factor 2

OX2R Orexin type 2 receptor

PGC-1 Peroxisome proliferator-activated receptor-γ coactivator 1

SOD2 Superoxide dismutase 2

TFEB Transcription factor EB

VPS34 Vacuolar protein sorting 34

## Introduction

The process of autophagy is a cellular regulation that involves the removal of dysfunctional or unnecessary components, such as damaged organelles and misfolded proteins. It allows cells to adapt to stress and renovate their cellular architecture. The process of elimination of harmful substances from the body, known as autophagy, can be used to treat various diseases, such as cancer and neurodegenerative disorders (Guo et al., 2018[26]). The models were able to identify the various interventions that can extend the lifespan of individuals by inducing autophagy. The accumulation of damaged organelles and macromolecules is the main cause of cellular aging (Vellai et al., 2009[81]). It occurs in long-lived post-mitotic cells. The decline in cellular repair and removal mechanisms is also related to the development of this condition. Studies have shown that the ability of the body to recycle nutrients through autophagy is a vital factor in regulating animal lifespan (Das et al., 2012[14]). One of the hallmarks of aging is the accumulation of damaged components, such as defective enzymes, DNA mutations, and proteinaceous aggregates (López-Otín et al., 2013[47]). Autophagy involves removing damaged components and digestion of intrinsic macromolecules. In addition to its role in meeting the cell's energy needs, the process of autophagy also helps to maintain the homeostasis of the organelle and prevents the development of age-associated processes (Yun and Lee, 2018[91]). It additionally contributes to the avoidance of proteotoxic stress and cytoprotection. The growing body of evidence supporting the link between aging and the process of autophagy has revealed the possibility of treating various age-related conditions. According to a study by Klionsky and colleagues (2021[38]), this pathway can prevent chronic low-grade inflammation and cellular aging. It involves the removal of damaged or dysfunctional components, such as organelles and protein aggregates. It helps in adaptation to stress, such as nutrient starvation and excessive reactive oxygen species production.

The goal of this review is to explore the various features of autophagy and how they can be linked to aging. It also highlights the recent discoveries that reveal the molecular mechanisms that underlie this phenomenon. Through studies on model organisms, we were able to identify the various hallmarks of aging, which are biological processes that become dysfunctional during an organism's lifespan. One of the most common signs of aging is the decline in certain genes' expression, like AMPK, IRE-1, etc. These genes are involved in the maintenance and development of protein homeostasis (Kikis et al., 2010[35]). In addition, studies on model organisms revealed that the various regulatory genes that are involved in the development and maintenance of protein homeostasis are also linked to lifespan extension (Hansen et al., 2018[27]). This review also highlights the various research opportunities that are available to study the link between aging and the development of this phenomenon. The various ways in which the decline of this process affects cellular health are explored. It also explains how this phenomenon can be linked to different age-related changes.

## Synopsis of Autophagy

Autophagy can be categorized into three main types: macro-, micro-, and chaperone-mediated autophagy. Both macro- and microautophagy can be categorized as non-selective or selective, and they have been best characterized in yeast. The most significant feature of macroautophagy is its ability to produce autophagosomes and double layer phagophores. In microautophagy, the cargo is sequestered through invagination or the protrusion or separation of the yeast membrane. The CMA process is mediated by the heat shock proteins, and these proteins bind to the substrates and deliver them to the lysosomes. Although non-selective autophagy is commonly used for the removal of bulk cytoplasm, selective autophagy is focused on the destruction of superfluous organelles, such as peroxisomes and mitochondria. Each process involves a core group of machinery and specific components, and the resulting process is referred to as mitophagy, xenophagy, pexophagy, etc. The development of membrane structures in autophagy is controlled by ATG genes, which are part of the conserved genes found in humans and yeast (Levine and Kroemer, 2019[41]). These genes can form complex structures that are regulated by the modifications that are carried out by the ATG proteins. The ATG proteins play a significant role in the various stages of the autophagic procedure, such as induction, nucleation, elongation, fusion, and degradation.

### Induction

The process of autophagy is initiated by the development of a phagophore within the membranes of different organs, such as the Golgi apparatus, ER, plasma membrane, and mitochondria. The ULK1 kinase complex, which includes the core components of ATG13, ULK1, and FIP200, is responsible for the induction of this process. In addition to ULK1, ULK2 can also play a similar function in the induction of autophagy.

### Nucleation

The catalytic subunits of the PI3K complex, which include PI3K, ATG14, VPS34, and BECN1, play a vital role in the nucleation process of phagophores (Chu et al., 2021[10]). One of the main functions of the PI3K complex's catalytic subunits is the production of phosphatidylinositol 3-phosphate. This substance serves as a major factor that influences the development of autophagosomes. Fan and colleagues (2011[18]) noted that ATG14 is a particular PI3K target that can maintain the membrane curvature of autophagosome membranes. In addition to promoting autophagy, the multifunctional protein BECN1 also controls the sensitivity of cells to death caused by apoptosis.

### Elongation

The phagophore can expand further following nucleation through the addition of a membrane protein, which can be achieved through ATG12-ATG5 and LC3I-LC3II conjugation systems (Romanov et al., 2012[64]). The ATG12-ATG5 conjugate can bind ATG16L1 in the phagophores. The latter forms are formed due to the stimulation of the autophagic response, which is required for the development of autophagosomes and the degradation of cargo.

### Fusion and degradation

Once the autophagosome has formed, its outer membrane is fused to lysosomes, which then produce autolysosomes. The cellular materials, such as ER and mitochondria, are destroyed by the enzymes in lysosomes. Although the exact factors that contribute to the fusion between lysosomes and autophagosomes are not known, the SNARE family is believed to play a significant role in the development of autolysosomes. Furthermore, the ATG14 protein can promote the fusion between lysosomes and the autophagosome by binding to the SNARE complex. This suggests that autophagy flux plays a vital role in the maturation and degradation of autophagosomes (Liu et al., 2015[45]).

## Role of Autophagy in Homeostasis and Cytoprotection

**Figure 1:**
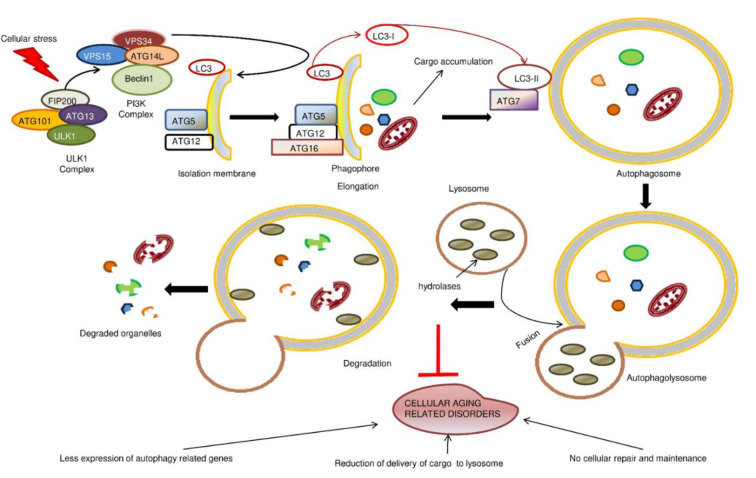
The mechanism of cellular autophagy and its relation to cellular aging-associated disorders

The continuous threat of exposure to xenobiotics and oxidative stress is a major factor that affects cellular survival and functionality (Pizzino et al., 2017[61]). The importance of autophagy is acknowledged, as it helps to maintain a balance between harmful and unnecessary molecules in the body and plays a vital role in maintaining healthy organelle homeostasis. The mechanisms that are involved in proteostasis activities are protein synthesis, protein degradation, and protein folding. These are all interconnected and have the potential to achieve robust stress responses. One of the most well-known pathways that is linked to the inhibition of autophagy is mTOR. It has been shown that mTOR's activation by the Nrf1 factor can inhibit the activity of the proteasome and balance its translational activity (Zhang and Manning, 2015[93]). In addition, the reduction of translation can help reduce the proteotoxic stress response. In addition to the ubiquitin system and molecular chaperones, autophagy is also a central component of cellular proteostasis (Margulis et al., 2020[52]). It can be triggered by the presence of a specific type of protein via CMA. The goal of this process is to remove bulk protein aggregates from the body through selective degradation. The collapse of protein homeostasis is a central feature of aging and age-associated diseases (Figure 1(Fig. 1)). It is often accompanied by the appearance of misassembled and mislocalized proteins. Lipophagy is a process that involves the degradation of lipids that can be caused by autophagosomes, which are involved in the accumulation of age-related protein aggregates (Schulze et al., 2017[70]). This process is an alternative method for organisms to manage their fat and regulate their homeostasis. Impaired lipophagy can lead to the accumulation of lipids, which can result in various health conditions such as hepatic steatosis and altered body mass (Carotti et al., 2020[5]).

ATG6, ATG9, and Beclin-1 are some of the cellular components that can lose their function due to age-related paralysis. In addition, these changes can decrease the lifespan of individuals. Similarly, in fruit flies, mutations in the core components of the autophagy system (ATG7 or ATG8) can increase the accumulation of insoluble proteins (Chang and Neufeld, 2010[6]). In addition, the knockout of ATG5 or ATG7 in mouse neurons can lead to the development of neurodegeneration and the appearance of various cytoplasmic inclusions in the brain (Sato et al., 2018[67]). On the other hand, the removal of the key receptor for the CMA in the liver, known as LAMP2A, can cause hepatic dysfunction and alter proteostasis. These aspects of aging are linked to the degradation and synthesis of proteins (Cuervo and Wong, 2014[13]). In mammals, the synthesis rates of proteins have reportedly gone down by up to 70 % during aging. Furthermore, studies have shown that proteostasis significantly declines in the aging process (Taylor and Dillin, 2011[79]). Animals are prone to accumulating damaged proteins, DNA, and organelles, which can be a sign of aging. It can also increase one's sensitivity to the harmful effects of damaging chemicals.

## Calorie Restriction - An Inducer of Autophagy

The three phases of the process of autophagy are regulation, phagophore formation, and degradation. The first step involves transferring extracellular signals to the core machinery of the autophagic body. The second step of the process involves the generation of double membrane phagophores that can be used to absorb cytoplasmic material. It is believed that these phagophores are produced from ER-mitochondria interfaces. ATG6 is an important component of the process that helps in the extension of the phagophore. It plays an important role in the integration between programmed cell death and autophagy (Hofius et al., 2011[30]). In addition to its binding partners, other ATG16L1 proteins also contribute to the stabilization of phagophores (Xiong et al., 2018[86]). Incipient phagophores are stabilized by ATG16L1 proteins, such as ATG12. The third step involves the degradation of cellular material. The vesicular autophagosome is also responsible for initiating this process by forming a lysosome-associated autophagosome combination. The ATG8 family proteins promote the loading of these autophagosomes by including LC3.

One of the most effective ways to induce autophagy is through the Calorie restriction (CR) strategy, which activates various regulatory pathways (Chung and Chung, 2019[11]). For instance, by inhibiting the activity of the TOR complex 1 (TORC1), CR triggers the activation of the ULK1 complex and AMPK, which are important components of the cellular transport machinery (Saxton and Sabatini, 2017[68]). In addition, the activation of CR by the SIRT1 protein helps to stimulate the production of essential autophagic proteins (Chung et al., 2010[12]). The growth signals generated by the cellular growth factor, which are receptor-bound to insulin-like growth factor, can also hinder the activity of autophagy via the Akt pathway (García-Mato et al., 2021[22]). According to Morselli et al., CR is a physiological inducer of the cellular process of autophagy. CR can extend the life span of a person by increasing the activity of SIRT1 (Morselli et al., 2010[56]). This enzyme can also act in the nucleus and cytoplasm. A variant of SIRT1 that is capable of inducing the degradation of various ATG gene products is as efficient as the wild-type variant. In addition to deacetylating various transcription factors, such as NF-kB, FOXO1, and p53, SIRT1 also exerts its effects on the regulation of life span (Yi and Luo, 2010[90]). The effects of CR on metabolic fitness and stress resistance are rapid. Emerging data suggest that these benefits can be applied to a wide range of conditions, making them potentially useful in clinical trials. However, it is generally considered unsuitable for use in combination with other drugs like wound healing and weight loss agents.

## Aging and its Detrimental Consequences

The concept of aging refers to a biological process that involves a gradual decline in the functioning of the body's cellular and functional components due to accumulated damage (Chowdhury et al., 2023[9]; Jin, 2010[31]). Although it is generally believed that aging is a multi-factorial process, various theories have been presented in an attempt to explain its age-related changes. It is known to cause various age-related conditions, such as cancer and neurodegenerative disorders. According to the aging theory, random damage accumulation can lead to various health issues. These include failure of the repairing capacity, which is mostly controlled by genetic and environmental factors (Rodríguez-Rodero et al., 2011[63]). For instance, the length of the telomere and the number of divisions that a cell can go through are some of the factors that regulate the process. According to the Free Radical theory, aging can lead to a reduction in the functioning of cells. This could be caused by the accumulation of reactive oxygen species. This theory has been supported by a body of evidence, as it suggests that aging leads to an increase in the production of ROS at cellular levels (Liguori et al., 2018[43]). It also suggests that a decline in the effectiveness of metabolic pathways is associated with this condition. Studies have also suggested that the CR mechanism could be used to offset the effects of aging on various cellular levels (Kim et al., 2020[36]). The development of epigenetic processes plays a role in reducing the rate of aging and lifespan. It is believed that the reduction in the IGF-1/insulin pathway can increase longevity (Vitale et al., 2019[84]). However, a study conducted by researchers led by Mercken and colleagues revealed that the CR process also resulted in a dramatic and uniform transcriptional reprogramming of human skeletal muscle (Mercken et al., 2013[54]). This resulted in a reversal of the cellular function that had been established to maintain and repair. Studies have shown that the decline in the AMPK pathway can lead to various cellular functions that are detrimental to one's health, while activation of the AMPK pathway can help slow down the aging process (Stancu, 2015[75]). AMPK's importance is acknowledged as a risk factor for developing age-related conditions. 

## The Entangled Relationship between Autophagy and Aging

The process of elimination known as autophagy is a complex process that involves multiple cellular components. To understand its characteristics in aging, researchers focused on stress factors and energy balance. The researchers noted that these factors play a role in the cell's decision-making processes (Sharifi-Rad et al., 2020[72]). This section will also cover the various pathways that regulate the development and maintenance of stress-related cellular responses. It is widely believed that the reduction in protein degradation via autophagy machinery accelerates the aging process (Gelino and Hansen, 2012[23]). Multiple studies have shown that the lack of this type of cellular protection could also cause various health conditions. These findings support the idea that functional autophagic systems are required for animal models to maintain their health span and normal lifespan. In humans, studies have shown that the expression of certain genes (ATG5, ATG7, and BECN1) that are involved in autophagy activities decreases with age (Aman et al., 2021[1]). It is also believed that the reduction in the delivery of cargo to lysosomes is a contributing factor to the development of age-related disorders. The evidence supporting the importance of autophagy in embryonic development comes from studies that use both laboratory and human samples. It shows that this cellular repair and maintenance process can help prevent age-associated inflammation (Glick et al., 2010[25]). Inflammation is a type of evolutionarily conserved mechanism that is designed to maintain homeostasis against injury or infection. It also serves as an adaptive response to these conditions.

**Figure 2:**
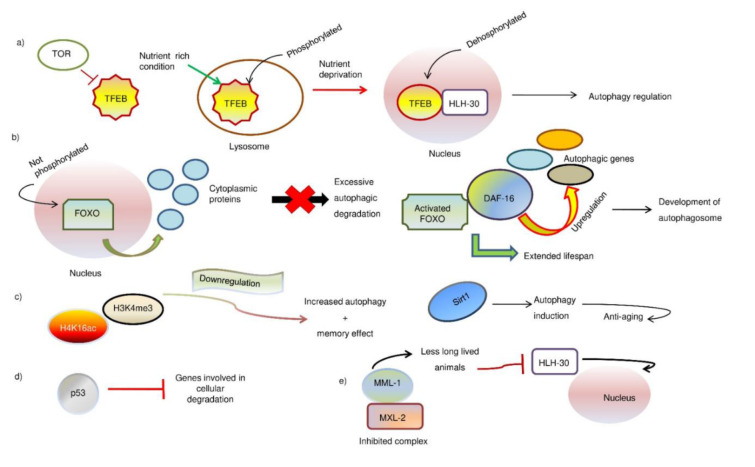
The different transcriptional and epigenetic factors associated with autophagy and aging. a) TFEB/HLH-30 mediated autophagy regulation. b) FOXO regulation in the induction of autophagy. c) Different histones and their roles in autophagy and aging. d) Role of p53. e) Role of MML-1/Mondo in the longevity of animals and consequent molecular effect

The lack of autophagic activity can accelerate aging because it could prevent the body from properly responding to age-associated cellular damage (Figure 2(Fig. 2)). Modifying the activity of pro-autophagy proteins in various species can extend the lifespan of animals. In mice, this can be done through the transgene-encoding of the ATG5 protein (Pyo et al., 2013[62]). *In vitro* studies conducted on aging revealed that overexpression of certain autophagy machinery components, such as ATG12 and ATG8, could extend the lifespan of the animals (Madeo et al., 2015[48]). The regulation and induction of the autophagic process are necessary for the maintenance of muscle strength and mass. This process can be activated by various pathways, such as those involving AMPK, PGC-1, and PI3K. The presence of certain compounds, such as mTOR inhibitors and AMPK activators, could modify this process. In addition, the activation of this process could suppress the development of low-grade inflammation. The activation of AMPK is also important in the induction of the autophagic process. This process controls the synthesis and degradation of the protein. AMPK can also phosphorylate the JNK1 and Bcl-2 pathways, which respectively trigger the dissociation of the Beclin-1-Bcl-2 complex and evoke the autophagic process (He et al., 2013[29]). The activation of the PI3k-Akt-mTOR pathway is also important in the development of autophagy processes. When the ULK1 complex is phosphorylated, the mTOR pathway is negatively involved in the development of this process (Yamasaki et al., 2020[89]). Studies have shown that certain molecules known as autophagic-related molecules can help to protect the genome from damage and oxidation. They also play a role in maintaining and repairing DNA. Paradoxically, the overexpression of the Bcl-1 gene in HeLa cells revealed that the activity of telomerase is decreased after the induction of autophagy (Taji et al., 2017[78]). The reduction in the length of telomeres can lead to genomic instability and contribute to age-related or cancer-related diseases. In addition, dysfunctional mitochondria can promote the accumulation of DNA damage and chromosomal alterations, which are also linked to the development of cancer cells. In this context, a lack of autophagy can lead to genome instability in mouse mammary epithelial cells. In artificial aneuploid mouse cells, the accumulation of autophagic molecules was shown to protect the cells from genome instability. 

## Aging is linked with Regulators and Down-Stream Effectors of Autophagy

### Protein regulators

#### AMPK

AMPK activation can lead to an increase in cellular energy levels and can also be triggered by various factors. For instance, by inhibiting the activity of the TOR pathway, AMPK can alter the expression of lysosomal and/or autophagy genes (Table 1(Tab. 1); References in Table 1: Eisenberg et al., 2009[17]; Fang et al., 2017[19]; Füllgrabe et al., 2013[21]; Giblin et al., 2014[24]; Johnson et al., 2014[33]; Liu and Xu, 2011[44]; Morselli et al., 2010[56]; Nakamura et al., 2016[58]; Ryu et al., 2016[65]; Slack et al., 2012[74]; Xu et al., 2019[87]; Zečic and Braeckman, 2020[92]). AMPK also phosphorylates PIK3C3, VPS34, and BECN1 (Kim et al., 2013[37]). These are important components of autophagic function. AMPK can either be regulated genetically or pharmacologically by metformin, which can help extend the lifespan of organisms (Slack et al., 2012[74]). The mTORC1 complex is a vital regulator of cell growth and degradation. The PIK3C3 and ULK1 kinase complexes are known to inhibit the activity of this protein. The TFEB phosphorylation also helps to block the protein's transcriptional activity. One of the most effective ways to prevent the development of age-related diseases is by reducing the mTOR pathway using rapamycin. It is believed that resveratrol, a known anti-aging agent, indirectly recruits AMPK through the activation of a protein kinase known as CAMKK2 (Lan et al., 2017[39]). This action, which is also known to exert its effects through Thr172 phosphorylation, is involved in the reduction of Aβ levels in neurons (Mairet-Coello et al., 2013[50]).

#### Sirtuins

However, this strategy does not eliminate the effects of other factors, such as the reduction in protein synthesis. In addition to this, the reduction in the number of mTOR signals also leads to a longer lifespan. According to Mercken et al. (2014[54]), SIRT1 is a deacetylase that can extend the lifespan of various organisms. SIRT1 is a known inhibitor of aging that can be used to extend the life spans of various animals, such as worms, flies, and yeast. It can also be pharmacologically activated or overexpressed. Histone deacetylation can be utilized by SIRT1 to regulate the expression of various genes involved in the process of autophagy (Sacitharan et al., 2020[66]). The interactions between SIRT1 and other molecules such as ATG5, LC3, and ATG7 can promote the deacetylation of these substances. They could be utilized to regulate the activity of various genes involved in the process of autophagy. It can also be pharmacologically activated or overexpressed. In addition, overexpression of SIRT6 can also improve the lifespan of these animals (Giblin et al., 2014[24]).

### Pharmacological regulators

#### Resveratrol

Resveratrol, a natural compound found in grapes, is known to activate the histone deacetylase sirtuin, known as SIRT1 (Xu et al., 2019[87]). In addition, resveratrol can also decrease the protein's acetylation. According to studies, the actions of resveratrol can increase the activity of the core proteins of the cell that are involved in the process known as autophagy (Josifovska et al., 2020[34]). This suggests that the compound can modify the acetylation state of the proteins through indirect or direct interactions. Although the regulation of the protein known as autophagy is important in the development and maintenance of disease and aging phenotypes, excessive amounts of it can also contribute to the cellular decline of aging. The ability of *C. elegans* to respond to the damage caused by autophagy plays a crucial role in how long it can live. But the effects of resveratrol on the lifespan of the animals that were treated with RNAi bec-1 are not so great (Hars et al., 2007[28]).

#### Spermidine

Spermidine is a natural polyamine that can extend the lifespan of several species, such as yeast, flies, and mice (Madeo et al., 2019[48]). Moreover, spermidine administration can improve the memory impairment experienced by flies. In flies, it can protect against this condition by altering the expression of certain markers of the degradation process. According to studies, the effects of spermidine on longevity and health were also influenced by the induction of autophagy (Eisenberg et al., 2009[17]). It can also help improve the survival of cultured human cells by increasing autophagy. Through the inhibition of histone acetylase activity, this chemical can epigenetically turn histone H3 into a hypoacetylated form (Burgio et al., 2016[3]). Various genes that degrade cellular components are also known to be affected by spermidine's impact on their expression. *In vitro* studies have shown that the effects of spermidine on the downstream mechanisms of lysosomal biogenesis and autophagy in mice are similar to those observed in humans (Ni and Liu, 2021[60]). Recently, a new pathway was discovered that allows spermidine to provide a residue for the translation of a proline motif in the eIF5a translation factor (Dever et al., 2014[16]). This motif is part of the master transcription factor of lysosomal biogenesis and autophagy, which is known as TFEB. In a study, it has been shown that treating old mice with a metabolite of spermidine can improve their levels of autophagy (Liao et al., 2021[42]). This finding suggests that the decline in endogenous spermidine can affect the level of this cellular process.

#### Rubicon

Recent evidence has shown that a decline in the role of the negative regulator of autophagy, known as the Rubicon, in adipocytes can exacerbate metabolic disorders (Minami et al., 2022[55]). The findings of this study indicate that the regulation of the process is important for the maintenance and development of age-associated phenotypes. In addition, it shows that excessive regulation of this process could contribute to the cellular dysfunction and functional decline of an organism (Yamamuro et al., 2020[88]).

#### Urolithin A

First-class compounds known to induce the mitophagy phenomenon are Urolithin A. According to a study conducted by Ryu and colleagues, urolithin A can prevent the development of dysfunctional mitochondria, which can extend the lifespan of *C. elegans* (Ryu et al., 2016[65]). The substance can prolong the normal activities of animals, such as mobility and pharyngeal pumping, while also maintaining the respiratory capacity of the mitochondria. In rodents, the effects of Urolithin A on the exercise capacity of older models of muscle function were demonstrated (Liu et al., 2022[46]).

#### Tomatidine

In older mice, a compound known as tomatidine can prevent skeletal muscle loss. It was found in unripe tomatoes. According to a study conducted on *C. elegans*, tomatidine can extend the lifespan and improve the health span of animals (Fang et al., 2017[19]). It also helps in various behaviors related to muscle health, such as swimming and pharyngeal pumping (Nakamura and Yoshimori, 2018[59]). The researchers noted that tomatidine can stimulate the production of ROS, which then activates various cellular antioxidant pathways (De Gaetano et al., 2021[15]). This process activates various cellular antioxidant pathways. It can also be observed in primary rat neurons and human cells.

### Transcriptional regulation of autophagy and aging

Post-translational modifications in the cytosol are the initial trigger of autophagy, which can also be regulated by various transcription factors. It is epigenetically regulated, and it is believed that prolonged exposure to autophagy helps to maintain the cell's flux while preventing it from becoming lethal. Different factors, such as the chromatin-modifying enzyme and the transcription factors, are responsible for regulating the activity of these genes, which are described below.

#### TFEB/HLH-30

TFEB is one of the regulators of the expression of lysosomes and genes related to autophagy. Overexpression of this factor has been shown to lengthen the lifespan of worms. It is known that the TFEB is negatively affected by the nutrient sensor mTORC1 (Settembre et al., 2012[71]). TFEB is a phosphorylated protein that is found on lysosomes in nutrient-rich conditions. After starvation, it is dephosphorylated and transferred to the nucleus, where its transcribed targets are located. In *C. elegans*, the homolog of the TFEB, HLH-30, is known to regulate the activities of autophagy and lysosomal genes (Lapierre et al., 2013[40]). This process is carried out by the inhibition of Insulin/IGF-related signaling. The conserved transcription factor HLH-30 is then transferred to the nucleus, where it is required for its longevity. Overexpression of the HLH-30 gene can extend the lifespan of wild animals (Nakamura et al., 2016[58]). The study revealed that the presence of the transcription factor TFEB and its related factor, HLH-30, are known to be important regulators of longevity. They are believed to be involved in the activities of several genes that play a role in lysosomes and autophagy (Franco-Juárez et al., 2022[20]).

#### FOXO

FOXO is a component that plays a role in the regulation of various genes that are related to autophagy and in the development of anti-aging interventions. It has been shown that overexpression of this factor can lengthen the lifespan of individuals (Murtaza et al., 2017[57]). When it's not phosphorylated within the nucleus, it interacts with a variety of proteins in the cytoplasm. This contributes to the negative feedback loop that prevents excessive autophagic degradation. In animals such as mice, the reduction of the activity of insulin-like growth factor-1 (IGF-1) can activate the FOXO/DAF-16 functions and extend lifespan (Zečić and Braeckman, 2020[92]). In worms, the presence of a protein known as DAF-16 upregulates some of the genes that are involved in the development of autophagosomes (Baxi et al., 2017[2]). Forkhead transcription factors, such as PHA4/FOXA, bind to the promoter's regions of Bec1 and Ulk1. These factors play a role in the development and activation of autophagosomes (Zhong et al., 2010[94]). The presence of these factors contributes to the development and activation of autophagosomes.

#### Histones

It was suggested that the regulation of autophagic processes could trigger a memory effect that could help speed up the response to starvation. In *C. elegans*, the reduction in the activity of H3K4me3 and H4K16ac was linked to the development of memory effects (Wells et al., 2012[85]). It is also believed that the downregulation of these two genes could be related to the induction of autophagy. The reduction in deacetylation is often linked to the development of increased autophagy (Füllgrabe et al., 2013[21]). This is because the H4K16 gene is involved in the transcription of various genes. The overexpression of Sirt1 has been regarded as a potential anti-aging intervention. It highlights the link between longevity and the induction of autophagy. It also stimulates the transcription of the various genes that are involved in the development of the cell cycle. Different stimuli can result in the deacetylation of H4K16 as well as the translocation of Atg8 and LC3 to the cytoplasm (Füllgrabe et al., 2013[21]). In addition, the presence of Sirt1 and its effects on the development of autophagy can be suppressed by certain epigenetic changes. These include the dimethylation of H3K9 and the silencing of histone H3K27 by the histone EZH2 (enhancer of Zest Homologue 2) (Jing and Lin, 2015[32]). H3K27 is also known to be associated with aging. In various species, including humans, the loss of this regulation was also observed.

#### p53

One of the most interesting factors that contribute to the development of age-related diseases is p53, a transcriptional regulator of the process known as autophagy (Shi et al., 2021[73]). In addition to promoting the growth of the cellular components, p53 also helps in the development of age-related diseases by inhibiting the accumulation of certain genes such as Nrf2 and SOD2 (Liu and Xu, 2011[44]). In the cytoplasm, p53 promotes the suppression of genes that are involved in the degradation of cellular components. 

#### MML-1/Mondo

The MML-1 and MXL-2 complexes are part of the Myc and Mondo families and play a role in the regulation of longevity in various organisms, such as *C. elegans* (Johnson et al., 2014[33]). They also play a role in the reduction of the generation of low-energy mitochondria and insulin signaling in *C. elegans* (Cheng et al., 2010[8]). The inhibition of the MXL-2 and MML-1 complexes can lead to the development of less long-lived animals that are unable to generate the HLH-30 nuclear localization (Nakamura and Yoshimori, 2018[59]). In some animals, these complexes preferentially regulate the various types of genes such as ATG2, ATG9, and Ulk1 that are involved in the development of autophagy.

### Insight of DNA damage as a chief source of autophagy and aging

The accumulation of damaged DNA and organelles is a common factor that contributes to aging. In some cases, accelerated aging is caused by a lack of repair enzymes. Bloom and Werner syndrome are examples of this. The two conditions are caused by mutations in BLM and the WRN genes, respectively (Schnabel et al., 2021[69]; Subramanian et al., 2021[77]). Another aging model that is lacking in ERCC6 or 8 is Cockayne Syndrome. A study conducted by Vermeij and colleagues revealed that defective DNA repair resulting from mutations in the Ku70, Ercc1, Ku80, and Xpd genes can lead to shorter lifespans in mice (Vermeij et al., 2016[82]). It was also known that the lack of DNA damage response components could contribute to the development of accelerated aging. These findings suggest that the impact of autophagy on the genomic integrity of cells could be significant. It was previously believed that the lack of ATG5 and beclin1 genes could cause genomic instability in the cells (Vessoni et al., 2013[83]). DSBs are made up of a combination of proteins, including the MRN and ATM kinases. When activated, these two components become auto-phosphorylated and bind to DSBs, which then leads to either HR repair or non-homologous end joining (Cannan and Pederson, 2016[4]). Removing HP1α from the DSB sites can trigger the development of Rad51 protein filaments. This is due to the presence of the RAD6 enzyme, which ubiquitinates the protein (Chen et al., 2015[7]). In addition, the enzyme's loosening of the chromatin can trigger the double-strand break repair process. The RPA, which is a replication protein A, recognizes single-strand breaks and recruits ATR, which promotes the phosphorylation of Chk1 and p53 as part of the response to irradiation. The initial events that lead to DDR is the activation of γH2AX by DNA-PKcs or ATM (Maréchal and Zou, 2013[51]). There are many intriguing connections between DDR and autophagy. The instability of the genomic structure of cells that are lacking in autophagy is linked to the compensatory upregulation of degradation, which results in reduced phosphor-Chk1 expression (Stead et al., 2019[76]). High levels of Chk1 are known to negatively impact genome integrity. This is because it is a component of the complex and a substrate for CMA. The importance of maintaining the correct outcome is maintained by carefully coordinating the activity of this enzyme with other proteins. In yeast, rapamycin-independent autophagy is known to be caused by DNA damage induced by various mechanisms, such as the activation of the DDR kinases Mec1, ATR, and Tel1 (Ueda et al., 2019[80]). One of the most interesting DNA repair links is the involvement of the p62 protein in the degradation of ubiquitinated components. Despite its role as a cytoplasmic process, the effects of autophagy on DNA repair can be numerous. For instance, moderate enhancement of DDR can have positive effects. The extension of the lifespan of long-lived mutators of the protein may depend on the development of DDR. However, due to the complexity of the DNA repair system, it is difficult to determine how this process can be improved.

## Conclusion and Prospects

Recent evidence supporting the link between aging and the development of autophagy has highlighted the importance of this process. It is believed that this process helps in the clearance of damaged organelles and proteins that are vital for the cellular homeostasis process. Studies in mice suggest that this process is a critical component of the aging process. Several studies published in recent years have focused on the relationship between the dysfunction of autophagy and the pathological consequences it can cause. The various age-related characteristics that are known to promote the development of cancer and neurodegenerative conditions are a gradual increase in the production of ROS, a decrease in DNA repair, and a progressive decrease in other proteostasis and antioxidant systems. Autophagy is also a vital part of the cellular homeostasis process. There have been numerous attempts to improve features related to aging, such as caloric restriction. Research has shown that limiting caloric intake can lead to various regulatory pathways that can promote the development of age-related conditions, such as cancer and neurodegenerative diseases. One of the most effective environmental interventions that can extend a person's lifespan is CR. Research has shown that restricting caloric intake can induce this process. It is believed that this environmental modification can help prevent aging. Although it is widely believed that the presence of autophagy can prevent aging, it is not yet clear how this can be achieved in a mechanistic manner. Further studies are needed to analyze the effects of epigenetic and post-translational changes on the regulation and function of this process. It's believed that the process of autophagy can promote cell maintenance by removing toxic components, suggesting that the upregulation of this process in long-lived cells could help them endure stressful conditions. It's believed that autophagy promotes longevity because it allows an organism to recover quickly from cellular damage. Although we can speculate that autophagosome selection is selective for certain cargos, it is not clear how this process might relate to aging. These gaps in knowledge suggest that we need to identify the key individuals who are involved in this process before we can fully understand its role in human aging. We have reviewed the various regulators and mediators of autophagy, which play a critical role in aging.

## Declaration

### Acknowledgments

The authors would like to acknowledge the academic fellowship grant from the Department of Science and Technology and Biotechnology, Govt. of West Bengal.

### Authors contribution

SGC performed the literature research and drafted the manuscript; RR prepared Figures 1-2, and PK participated in modifying the article. All authors reviewed the manuscript.

### Funding

This work is financially supported by the Department of Science and Technology and Biotechnology (Sanction no. BT/ST/P/S&T/ 2G-13/2017), Govt. of West Bengal.

### Data availability

Not applicable.

### Conflict of interest

The authors declare that they have no competing interests.

### Ethical approval and consent to participate

Not applicable.

### Consent for publication

All authors approved the publication.

## Figures and Tables

**Table 1:**
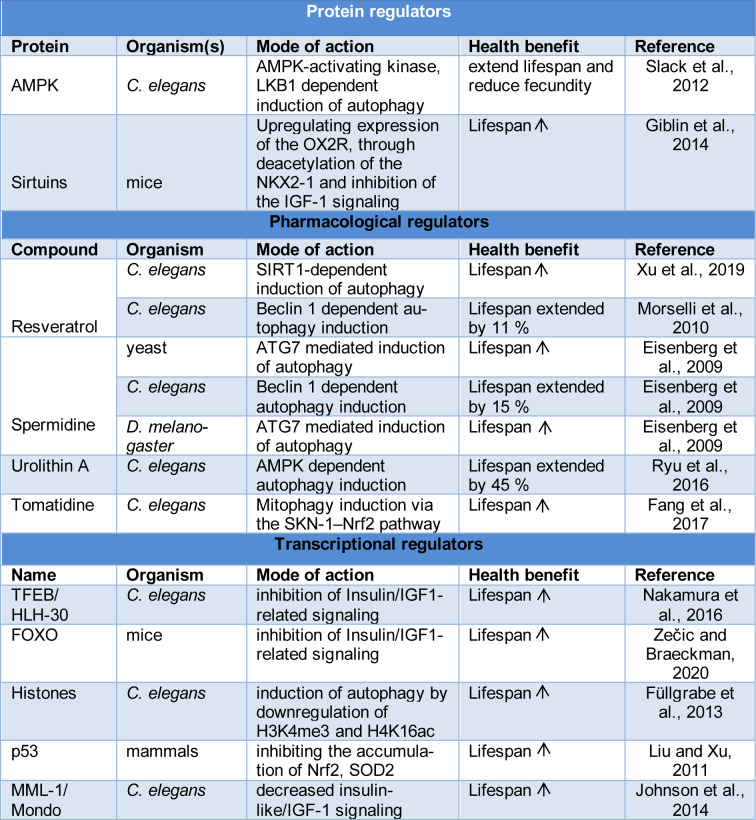
List of Autophagy regulators involved in longevity and anti-aging
